# Morphometric Differences in Planum Temporale in Schizophrenia and Bipolar Disorder Revealed by Statistical Analysis of Labeled Cortical Depth Maps

**DOI:** 10.3389/fpsyt.2014.00094

**Published:** 2014-08-01

**Authors:** J. Tilak Ratnanather, Shannon Cebron, Elvan Ceyhan, Elizabeth Postell, Dominic V. Pisano, Clare B. Poynton, Britni Crocker, Nancy A. Honeycutt, Pamela B. Mahon, Patrick E. Barta

**Affiliations:** ^1^Center for Imaging Science, Johns Hopkins University, Baltimore, MD, USA; ^2^Institute for Computational Medicine, Johns Hopkins University, Baltimore, MD, USA; ^3^Department of Biomedical Engineering, Johns Hopkins University, Baltimore, MD, USA; ^4^Department of Mathematics, Koç University, Istanbul, Turkey; ^5^Department of Psychiatry, Johns Hopkins University School of Medicine, Baltimore, MD, USA

**Keywords:** cortical mantle, planum temporale, schizophrenia, bipolar disorder, cortical thickness

## Abstract

Differences in cortical thickness in the lateral temporal lobe, including the planum temporale (PT), have been reported in MRI studies of schizophrenia (SCZ) and bipolar disorder (BPD) patients. Most of these studies have used a single-valued global or local measure for thickness. However, additional and complementary information can be obtained by generating labeled cortical distance maps (LCDMs), which are distances of labeled gray matter (GM) voxels from the nearest point on the GM/white matter (WM) (inner) cortical surface. Statistical analyses of pooled and censored LCDM distances reveal subtle differences in PT between SCZ and BPD groups from data generated by Ratnanather et al. (Schizophrenia Research, http://dx.doi.org/10.1016/j.schres.2013.08.014). These results confirm that the left planum temporale (LPT) is more sensitive than the right PT in distinguishing between SCZ, BPD, and healthy controls. Also confirmed is a strong gender effect, with a thicker PT seen in males than in females. The differences between groups at smaller distances in the LPT revealed by pooled and censored LCDM analysis suggest that SCZ and BPD have different effects on the cortical mantle close to the GM/WM surface. This is consistent with reported subtle changes in the cortical mantle observed in post-mortem studies.

## Introduction

Recent studies suggest that thicknesses of cortical structures seen in MRI scans may be different in patients with neurodevelopmental diseases, such as schizophrenia (SCZ) and psychotic bipolar disorder (BPD), and that these changes may be related to genetic and environmental factors (1–6). In particular, differences in cortical thickness have been associated with abnormalities in the posterior or caudal superior temporal gyrus (STG), part of which includes the planum temporale (PT). The PT is a multisensory region on the ventral surface of the STG and plays an important role in language and speech processing (7). These functions may be disrupted by auditory hallucinations resulting in the classic psychotic symptoms of SCZ (8), which differ from those seen in BPD (9). Thus, the PT continues to be important in neuroimaging studies of SCZ.

One method of quantifying cortical thickness is labeled cortical distance mapping (LCDM), which analyzes a subvolume encompassing the region of interest (ROI). The approach requires precise definitions of anatomical boundaries, which can be compounded by interindividual variability. The boundary between white matter (WM) and CSF is often indistinct, but this challenge can be partially overcome by viewing the ROI as a laminar structure composed of gray matter (GM) voxels and a local surface coordinate system based on an anatomically defined GM/WM cortical surface (10). This approach is in line with the classic formulation of differential geometry, in which local coordinate systems are examined. In this case, the LCDM data are represented by a set of distances from labeled GM voxels with respect to the nearest point on the GM/WM surface.

There are several ways of analyzing LCDM data. The simplest is to use the 95th percentile of the LCDM as the overall thickness, a definition, which has been used in several neuroimaging studies of SCZ in the cingulate (11, 12), prefrontal cortex (13), and parahippocampal gyrus (14). A second approach is Local LCDM, which condenses to a single distance value at each point on the surface. Local LCDM was used to examine variation in thickness across cingulate (15) and the left PT (16) surfaces in SCZ. In the latter study, the variation in thickness was found to be consistent with post-mortem analysis (17). Finally, a third approach is to define a “laminar thickness” as the number of voxels at the 95th percentile LCDM, divided by surface area. This was used in an expansion of the Qiu et al. (16) study and revealed gender and laterality effects on the PT, with reduced laterality found in SCZ but not BPD (18). Approaches similar to LCDMs have also been developed and used in other studies (19–26).

This paper describes the use of a fourth approach, namely pooled and censored LCDM analysis, that can offer further information than just summary measures of thickness and volume. For example, it is possible to pool the LCDM distances for each diagnostic group and perform statistical comparisons on the overall distance level for each group, rather than the individual level; further, it is possible to censor these distances to compare the diagnostic groups at specific distances from the GM/WM surface (27, 28). This is achieved by merging the distances for subjects from the same group to utilize more of the information provided in these distances. The other three methods use summary measures based on LCDM distances and tend to over summarize the information contained in these distances. Hence, group differences in tissue morphometry might be missed. In this fourth method of pooled and censored LCDMs, the common group morphometric traits are emphasized and the effect of individual variation is lessened. For example, pooling appears to be effective even with small to medium sample sizes (i.e., number of cases and controls) in structures known to be affected by disease such as the cingulate in dementia (29) and deficit SCZ (30). Thus, we aim to demonstrate that applying the pooled and censored LCDM technique to the Ratnanather et al. (18) dataset offers an alternative and interesting way of quantifying differences in the effect of SCZ and BPD within the PT cortical mantle.

## Methods

### Data

**Figure 1:**
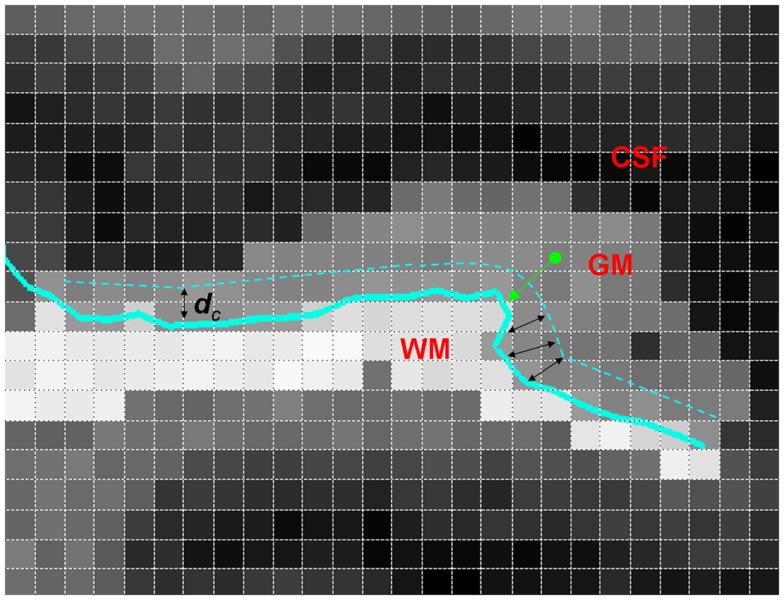
**A two-dimensional illustration of LCDM distance computation (i.e., the normal distance) from a GM voxel to the GM/WM PT surface (thick green arrow)**. Also shown is the censoring procedure with censoring distance *d*_c_ (black double arrows). At this censoring step, the GM voxels with centroid closer to the GM/WM surface than *d*_c_ are retained.

Details of subjects, MRI acquisition, segmentation, and GM/WM surface reconstruction are described in Ratnanather et al. (18). A brief description follows. Using a 1.5-T scanner, T1 scans with 1 cm^3^ isotropic resolution across the entire cranium were obtained for 94 subjects grouped on age, sex, and ICV. Study groups included healthy controls [*n* = 27, 39.9 ± 11.1 (mean ± SD) years old, 52.8% male], SCZ (*n* = 31, 41.4 ± 9.5 years old, 54.8% males), and BPD (*n* = 36, 44 ± 15.6 years old, 44.4% males). Bayesian segmentation of ROI masks for the STG was used to classify image voxels as GM, WM, or CSF. The GM/WM threshold from the segmentation was used to generate triangulated isosurfaces representing the 2D cortical surface. The subsurface corresponding to the PT was extracted by cutting the triangulated surface via curvature-based dynamic programing delineation of gyral and sulcal boundaries (31). The rules first developed and validated for the PT were used with one exception. Namely, the anterior boundary was delineated by tracking from the retroinsular end of Heschl’s Sulcus (HS) to the STG. GM voxels closer to the PT overlapping surface were included in the statistical analyses. In order to get a distance map for the GM, the distance between the centroid (center of mass) of each GM voxel and the closest GM/WM surface vertex was calculated, with 1 mm × 1 mm × 1 mm resolution. These data give information on the probability distribution of the GM distance from the GM/WM surface. The PT surface provides the natural local coordinates associated with the GM/WM surface. The third dimension is described by the normal coordinates measuring the distance of the GM voxel from the surface yielding LCDMs. Figure 1 illustrates the computation of distances for the LCDMs.

### Statistical analysis

#### Pooled LCDM

Following Ceyhan et al. (27), the LCDM distances were combined across subjects within each diagnostic group, resulting in a set of distances called pooled distances. Distances were restricted to the range of −1 to 4.5 mm for anatomical reasons (17). This resulted in an average of 0.42 and 0.50% of distance values being discarded from the left planum temporale (LPT) and RPT data, respectively. The pooled LCDMs were statistically compared using non-parametric tests against the null hypothesis that they come from the same distribution. Kruskal–Wallis (KW) tests were used for the three-group analyses. Any significant results were followed-up using Mann–Whitney *U* (MWU) tests, Wilcoxon Rank Sum tests, and Kolmogorov–Smirnov (KS) tests for pairwise comparisons as *post hoc* tests, comparing all pairs of diagnostic groups. Each test was initially two-sided. If significant, one-sided (i.e., greater than and less than versions of the) tests were performed to determine the direction of significant difference. Holm’s correction was applied to *p*-values to account for the multiple pairwise comparisons between the diagnostic groups (32).

In this method of pooled distances, between-sample independence appears to hold (i.e., pooled LCDM distances between the diagnostic groups can be assumed to be independent); however, within-sample independence is violated as the distances for each individual are not independent. This dependence arises from voxels of the same subject having spatial dependence on nearby voxels. Although pooling does not mitigate this dependence problem, it has been shown that its influence is negligible when employing non-parametric tests based on ranking of the distances (27). Despite this assumption violation, a major advantage of our method is the ability to use all of the information provided by LCDM distances. The fact that the sample sizes are large is for the most part not causing spurious significance. Although the pooled distances emphasize the main traits, individual variations in morphometry provide sufficient noise to counterbalance the effects of very large number of data points.

#### Censored LCDM

Following Ceyhan et al. (28), the pooled LCDM distances were tested at distances increasing by fixed increments of 0.01 mm. That is, for the first censoring step, pooled distances ≤0.01 mm were tested for group differences; at the second step, distances ≤0.02 mm were tested; and so forth until the maximum distance was reached (where at this last stage, censored distance analysis is equivalent to the pooled distance analysis). Figure 1 illustrates censoring at the threshold distance *d*_c_ where the GM voxels whose centroids are closer to the GM/WM surface than *d*_c_ are retained for analysis at that censoring step. The *p*-values were computed at each censoring step for the KW and MWU tests. Again Holm’s correction was applied to *p*-values to account for the pairwise comparisons.

Censored LCDM distances inherit the strengths and weaknesses of the pooled LCDM distances. In particular, the problem of within-sample dependence at each censoring step persists, but this was again shown to be negligible (28). On the other hand, censoring provides a powerful methodology that can identify at which distance values (with respect to the GM/WM surface) the potential differences start to occur. Thus, it provides information that is not available from the pooled distances. One further note is that, by construction, KS tests are not meaningful for censoring distance analysis and therefore were not performed on the censored distances.

## Results

**Figure 2:**
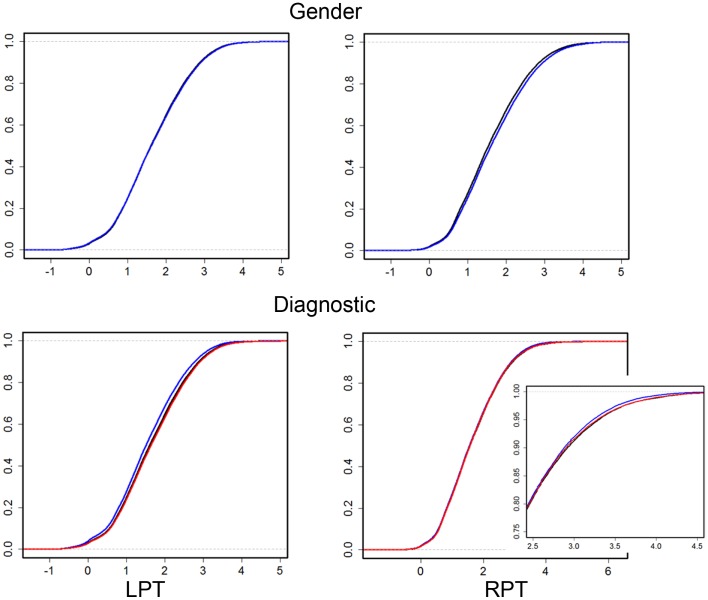
**Empirical cumulative distribution function (ECDF) plots of pooled LCDMs for LPT (left column) and RPT (right column)**. Top row: male (blue) and female (black). Bottom row: control (black), SCZ (blue), and BPD (red). Bottom right shows a zoomed plot for better visualization. The abbreviations are as in Table 1.

**Figure 3:**
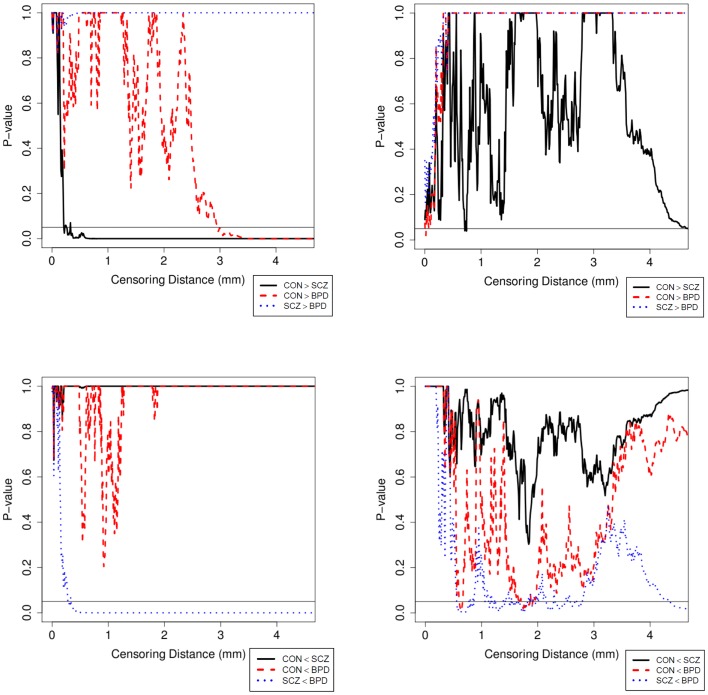
**Plot of *p*-values for the MWU test between diagnostic groups in the LPT (left column) and RPT (right column)**. *p*-Values are adjusted with Holm’s correction for multiple comparisons between the diagnostic groups. The abbreviations are as in Table 1.

Results were obtained with statistical significance level set at 0.05. Figure 2 shows the empirical cumulative distribution function (ECDF) plots of pooled LCDMs for the LPT and RPT; the small negative distances reflect the sub-voxel inaccuracy of the generated surface. Multi-group comparisons for the two structures revealed overall differences between groups (KW, *p* < 0.001 for all except *p* = 0.0098 for RPT). Table 1 shows *p*-values for the MWU tests for pairwise comparisons of diagnostic groups and of gender. Table 2 shows *p*-values for KS tests for pairwise comparisons of diagnostic groups. Table 3 shows *p*-values for KS tests for pairwise comparisons of gender. Table 4 shows the results from the MWU tests of censored distances with significant *p*-value cutoffs for LPT and RPT, by diagnosis. Also shown are the results of the KW tests for all groups. Table 5 shows the censored distance analysis for the PT by gender. Figure 3 shows *p*-value plots of censored analysis of the LPT and RPT by diagnosis, based on the MWU test.

**Table 1:** **Comparison of thickness (mm) in diagnostic groups and gender with pooled LCDM analysis**.

	MWU *p*-value	Conclusion
CON vs. SCZ
LPT	<0.001	CON thicker than SCZ
RPT	0.0312	No significance
CON vs. BPD
LPT	<0.001	CON thicker than BPD
RPT	0.8444	No significance
SCZ vs. BPD
LPT	<0.001	SCZ thinner than BPD
RPT	0.0208	No significance
M vs. F
LPT	0.1762	No significance
RPT	<0.001	Males thicker than females

*CON stands for controls, SCZ for schizophrenia, BPD for psychotic bipolar disorder, MWU for Mann–Whitney *U* test, LPT for left planum temporale, RPT for right PT. M and F stand for males and females, respectively*.

**Table 2:** **Kolmogorov–Smirnov (KS) test for ECDF comparisons of pooled LCDM distances in diagnostic groups**.

	KS test			Conclusion
	Two-sided	1st ECDF < 2nd ECDF	1st ECDF > 2nd ECDF	
CON vs. SCZ				
LPT	<0.001	<0.001	0.9999	CON thicker than SCZ
RPT	0.1473	0.1715	0.9999	No significance
CON vs. BPD				
LPT	<0.001	<0.001	0.9999	CON thicker than BPD
RPT	0.6314	0.9999	0.9999	No significance
SCZ vs. BPD				
LPT	<0.001	0.9999	<0.001	SCZ thinner than BPD
RPT	0.1473	0.9999	0.2415	No significance

*Other abbreviations are as in Table 1*.

**Table 3:** **KS test for ECDF comparisons of pooled LCDM distances in gender groups**.

M vs. F	KS (two-sided)	KS (<)	KS (>)	Conclusion
LPT	0.0022	0.0011	0.4184	Males thicker than Females
RPT	<0.001	<0.001	0.9999	Males thicker than Females

*Abbreviations are as in Tables 1 and 2*.

**Table 4:** **Censored significant *p*-value cutoffs for LCDM distances compared by diagnosis**.

Multi-group comparison by KW test			
LPT	(0.35,4.5)
RPT	–

**Pairwise comparisons by MWU test**			

**Comparison**	**ROI**	**Direction**	
		**1t^st^ > 2d^nd^**	**1t^st^ < 2d^nd^**

CON, BPD	LPT	(1.89,4.5)	–
	RPT	–	–
SCZ, BPD	LPT	*–*	(0.35,4.5)
	RPT	*(4.35,4.5)*	–
CON, SCZ	LPT	*(0.22,4.5)*	–
	RPT	*(4.28,4.5)*	–

*Numbers listed are the ranges of distances (in millimeters) at which the null hypothesis is rejected. Numbers are in italics when this value is lower than the distance at which the multi-group comparison’s null hypothesis is rejected, suggesting that they may not be meaningful below the multi-group comparison significance threshold. ROI stands for region of interest; the other abbreviations are as in Table 1*.

**Table 5:** **Censored significant *p*-value regions for the LCDM distances compared by gender**.

ROI	Direction	
	Male > female	Male < female
LPT	(1.35,2.60)	–
RPT	–	[0.88,4.00]

*Results are obtained from the MWU test. Numbers listed are the ranges of thickness (millimeters) in which the null hypothesis is continually rejected. The abbreviations are as in Table 1*.

## Discussion

This paper describes the application of non-parametric statistical methodology to provide a different and complementary perspective of LCDMs with reference to the PT in a differential analysis of SCZ and psychotic BPD in a medium sized population. The three non-parametric statistical tests (i.e., KW, MWU, and KS) were chosen because the distribution of LCDM distances is clearly non-Gaussian. All three tests are sensitive to differences in the distribution of LCDM distances but provide different aspects of the information conveyed by LCDM distances with certain limitations. Conceptually, the KW test is an omnibus test indicating whether distance distributions among two or more groups are different or not, while MWU tests reveal the pairwise group differences. On the other hand, KS tests may provide stochastic ordering (when used together with the corresponding ECDF plots). Given that the KW test shows an overall group difference, the MWU tests yield information about a more biologically meaningful question: “on average, do distances in one group tend to be less than in another group?” A significant MWU test for a left-sided alternative comparing the distances for two groups, say A and B, would imply that a randomly chosen cortical GM voxel tends to be located closer to the GM/WM boundary in group A than in group B (which suggests cortical thinning in group A is more likely). The KS test, like the KW test, tests for differences in the distribution between groups. Unlike the KW test, the KS test can also be adapted to check for stochastic ordering. The usual KS test depends on the largest *vertical* distance between two ECDFs. The one-sided version of the KS test depends on the largest *vertical* distance between these distributions. Although MWU and KS tests both answer distributional questions, they assess different aspects of the distributional structure. In particular, the MWU test provides differences in the distribution of the ranked distances, while the KS test provides differences in ECDF estimates. Furthermore, the MWU test is complementary, in the sense that it can only be significant for one one-sided alternative and the uncorrected *p*-values for both one-sided alternatives (i.e., left and right side) add up to 1. In contrast, the KS test may be significant for both left- and right-sided alternatives, occurring at different distance values. Also, the KS test will almost never be complementary for one-sided tests. Practical and theoretical aspects of pooled and censored LCDM analysis have been discussed extensively elsewhere (27, 28). In particular, Monte Carlo simulations suggest that these approaches are capable of capturing differences between diagnostic groups. Furthermore, it is emphasized that censoring should be understood in a pointwise fashion, not as testing a whole range of LCDM distances (28).

Our results confirm that the LPT is more sensitive than the RPT in distinguishing between SCZ, BPD, and healthy controls (Tables 1 and 2; bottom row of Figure 2). Also confirmed is a strong gender effect, with males displaying a thicker PT than females (Table 3; top row of Figure 2). The differences between groups at smaller distances in the LPT suggest that SCZ and BPD have different effects close to the GM/WM surface. This suggests the presence of subtle changes in the cortical mantle (Figure 3). These differences are consistent with those reported elsewhere. For instance, reduced lateralization correlated with increased severity of symptoms, suggesting that laterality is a biological risk for SCZ (33).

At the macroscopic level, pooled and censored analysis of LCDMs may offer a perspective of changes within the cortical mantle. Granted that a flat cortex would result in a top-hat like LCDM profile and a non-uniform distribution of thickness over the cortical region would result in a skewed LCDM profile, it is not surprising that in view of classical differential geometry (34) the LCDM shape is influenced by local curvature or gyrification and thickness. Additionally, the mantle can be viewed as an aggregation of cortical columns whose orientation and spacing contribute to local cortical thickness and curvature (35) and gyrification (6). Thus, macroscopic changes reflected by differences in LCDM shape could be attributed to changes at the microscopic (i.e., cellular) level within these cortical columns.

At the microscopic level, the cortical mantle comprises six cortical layers that are numbered I–VI as one goes from the outer or pial (i.e., GM/CSF) boundary away from the skull inwards to the GM/WM surface (36). Each layer is thought to comprise different cells such as neuronal, pyramidal, non-pyramidal, and glial cells that are important in neurotransmission between the different layers as well as with other cortical and subcortical regions (36). Estimates of neuronal and glial densities in different cortical regions have been obtained from several histopathological studies in humans and mammals. Altered measures have been suggested as explanations for cortical thinning observed at the macroscopic level in other neuroimaging studies (37, 38).

There have been a few histopathological studies of SCZ and BPD in the PT in humans (39–42). Beasley et al. (39) observed reduced neuronal clustering in BPD and SCZ together with reduced neuronal size in layer III of the PT; they suggest that the subtle alterations may be due to neuronal organization within mini-columns or within layers. Smiley et al. (40, 42) suggest that reduction in neuron density and volume of layers I–III (deep or infra-granular) may affect differences in LPT in SCZ and that thinner upper layers disrupt multisensory integration and phonetic processing as seen in SCZ. Simper et al. (41) suggested that layer III cell density and pyramidal neuron size differences in the PT are associated with callosal WM abnormality in SCZ, which may affect laterality. Based on these histopathological studies, it is conceivable that differences in LCDMs at distances from the GM/WM surface (Tables 4 and 5; Figure 3) may be characterized by corresponding density changes. For example, reduced density could result in thinner layers and thus altered cortical columns. However, no definitive conclusion can be reached until LCDM analysis can be correlated with measures in histopathological MRI studies of SCZ [e.g., Ref. (38)].

In summary, pooled and censored LCDMs offer alternative and detailed analyses of distances of GM voxels relative to the cortical surface. Further understanding of the biological implications of pooled and censored LCDMs will be necessary. One approach would be to use LDCMs to correlate ante-mortem or post-mortem MRI analyses with histological sections. This would allow for understanding how analysis of LCDM shape is influenced by macroscopic and microscopic changes. Finally, it is affirmed that the specificity of the PT in SCZ suggests that the PT is a contributor to structural abnormalities associated with functional abnormalities in SCZ.

## Conflict of Interest Statement

The authors declare that the research was conducted in the absence of any commercial or financial relationships that could be construed as a potential conflict of interest.
